# Revisiting the gravity laws of inter-city mobility in megacity regions

**DOI:** 10.1007/s11430-022-1022-9

**Published:** 2022-12-22

**Authors:** Pengjun Zhao, Haoyu Hu, Liangen Zeng, Jun Chen, Xinyue Ye

**Affiliations:** 1 College of Urban and Environmental Sciences, Peking University, Beijing, 100871 China; 2 School of Urban Planning and Design, Peking University Shenzhen Graduate School, Shenzhen, 518055 China; 3 Laboratory for Laboratory for Earth Surface System and Human-Earth Relations, Ministry of Natural Resources of China, Peking University Shenzhen Graduate School, Shenzhen, 518055 China; 4 National Geomatics Center of China, Beijing, 100048 China; 5 Department of Landscape Architecture and Urban Planning, Texas A&M University, College Station, TX 77843 USA

**Keywords:** Mobility, Inter-city, Travel behaviour, Megacity region, Zipf’s law, China

## Abstract

Inter-city mobility is one of the most important issues in the UN Sustainable Development Goals, as it is essential to access the regional labour market, goods and services, and to constrain the spread of infectious diseases. Although the gravity model has been proved to be an effective model to describe mobility among settlements, knowledge is still insufficient in regions where dozens of megacities interact closely and over 100 million people reside. In addition, the existing knowledge is limited to overall population mobility, while the difference in inter-city travel with different purposes is unexplored on such a large geographic scale. We revisited the gravity laws of inter-city mobility using the 2.12 billion trip chains recorded by 40.48 million mobile phone users’ trajectories in the Jing-Jin-Ji Region, which contains China’s capital Beijing. Firstly, unlike previous studies, we found that non-commuting rather than commuting is the dominant type of inter-city mobility (89.3%). Non-commuting travellers have a travel distance 42.3% longer than commuting travellers. Secondly, we developed more accurate gravity models for the spatial distribution of inter-city commuting and non-commuting travel. We also found that inter-city mobility has a hierarchical structure, as the distribution of inter-city travel volume follows Zipf’s law. In particular, the hierarchy of non-commuting travel volume among the cities is more in line with an ideal Zipf distribution than commuting travel. Our findings contribute to new knowledge on basic inter-city mobility laws, and they have significant applications for regional policies on human mobility.

## 1. Introduction

A megacity region is a highly urbanised area in which dozens of cities including megacities are located, connect, interact and integrate. It is an advanced form of human urban settlement. It is projected that there will be 43 megacities, and 68% of the world's population will live in megacity regions by 2050 (United Nations, 2019). Although the existing megacity regions are mostly in Europe and North America, Asia has contributed enormously to global urban population growth in megacity regions in the past few decades. For example, in China's Jing-Jin-Ji Region, the population grew from 69.8 million in 2008 to 111 million in 2020.

Inter-city mobility plays an important role in megacity regions' sustainable development. It is essential for millions of residents to access the regional labour market, goods and services. Inter-city mobility is also a key issue in relation to the spread of infectious diseases, such as COVID-19 (Gibbs et al., 2020; Pan et al., 2020; Unwin et al., 2020). Some important laws of human mobility have been proposed based on the existing research. First, human trajectories show a high degree of temporal and spatial regularity (González et al., 2008). Each individual is characterised by a time-independent characteristic travel distance and a significant probability of returning to a few highly frequented locations. Second, human travel on geographical scales is an ambivalent and effectively superdiffusive process (Brockmann et al., 2006). The distribution of traveling distances usually decays following a power law ($P(r)\sim r^{-(1+\beta)}$). Third, the travel volume of inter-city mobility follows the gravity model ($V=kO^{\alpha}D^{\beta}/c^{\theta}$), which means the volume is positively correlated with the population or economy size of the cities, but inversely correlated with the travel costs between cities (Zipf, 1946; Shen, 2004). Fourth, research has shown that human mobility has a hierarchical structure (Bassolas et al., 2019; Alessandretti et al., 2020; Bettencourt and Zünd, 2020). A hierarchical structure of mobility can exist on both intracity and inter-city scales. The population of a city and its rank in a size table usually follow Zipf's law ($\text{Rank}^{\gamma}P^{\delta}=a$) (Zipf, 1940), which is also used to fix the frequencies of individual travel destination choices (Yan et al., 2017).

However, our existing knowledge of inter-city mobility laws needs further improvement for three reasons. Firstly, the current conclusions are mainly drawn from large cities, while evidence from megacity regions with more than 100 million people and large geographics scale is scarce. Megacity region is a new form of urban space, which is a dense, urbanized region with multiple sizes of cities, proximately located and functional networked, clustered around one or two large cities (Hall, 2009). Inter-city mobility in megacity regions is different from that in other regions in that boundaries between urban and rural areas tend to be blurred and that socioeconomic ties of various types are more complex and intertwined. Many studies have found that regional population size, geographical scale and distance between potential origin and destination locations are significant factors influencing people's travel behaviour and, thus, inter-city mobility (Barbosa et al., 2018; Liang et al., 2013; Noulas et al., 2012; Toch et al., 2019; Yan et al., 2013). Therefore, there is a strong need to determine inter-city mobility laws in megacity regions to improve our existing inter-city mobility knowledge. Secondly, the existing laws do not account for the diversity of travel purposes, as they are mainly focused on commuting (Suh, 1988), while few research studies have paid attention to non-commuting inter-city travel. Non-commuting travel, which includes all travel behaviours besides commuting, such as shopping, tourism and medical treatment, is important for the quality of life (Lannoo et al., 2018). Therefore, non-commuting could be a nonnegligible part of inter-city mobility. Thirdly, the existing evidence is mainly based on traditional data, such as travel survey data, road traffic data, etc. These data have some weaknesses (Ashtakala and Murthy, 1993), for instance, collecting travel survey data is expensive, the data cannot reveal real-time travel patterns over a long period and the sampling rate is small. These weaknesses become more obvious in a megacity region with more than 100 million residents. Big data offer advantages over conventional data sources in terms of volume, velocity, variety and veracity (Yaqoob et al., 2016). Several types of big data are often used, for example, mobile phone data (Alexander et al., 2015; Zhong et al., 2016), smartcard record data (Faroqi et al., 2018; Medina, 2018) and social media data (Yang et al., 2019). Mobile phone trajectory big data have the advantage of showing real-time information on the movements of a large population with high spatial accuracy and wide geographical coverage (Nitsche et al., 2014; Steenbruggen et al., 2015). We use mobile phone trajectory big data to analyse inter-city mobility patterns and reverify gravity laws of it.

The main research questions of this paper are as follows. First, what are the differences between inter-city commuting and inter-city non-commuting travel behaviours in the megacity region? Second, what determines the distribution of inter-city mobility in the megacity region? We try to optimise the gravity model to depict the relationship between inter-city mobility and the regional urban system, and we examine the gravity model's validity for different travel purposes. Third, does the volume of inter-city mobility have a typical hierarchical structure? There has been wide discussion of applying Zipf's law to the city population, but it has rarely been examined regarding mobility. We inspect and contrast Zipf's law with population distribution.

## 2. Data and methods

### 2.1 Empirical datasets

The mobile phone data used in this paper came from one of the three largest telecom companies in China. The data were collected in the first week of June 2018 in Jing-Jin-Ji. The company has a market share of 27% of all mobile phone users in this region. This region has a land area of 217,000 km$^2$ and a population of 110.9 million. It covers 13 prefectures and province-level municipalities, including Beijing, the capital of China. This region covers hundreds of cities around Beijing and is a typical megacity region in China.

Data used in this research are aggregated according to time, space and user attributes to protect personal information privacy. The time interval of data recording is 0.5 h, and the spatial unit of data is a 1 km grid. Table 1 gives an example of the mobile phone data for this research. We examined the thoroughness of the mobile phone data and cal-

**Table 1** Example of original mobile phone data

| Mobile Phone ID | Location Cell ID | Latitude | Longitude | Timestamp |
|---|---|---|---|---|
| 1018566A2323B212C | 3003403382115386 | 39.959644°N | 116.097512°E | 20180603123735 |
| 1018566A2323B212C | 3003403474115334 | 39.899247°N | 116.233285°E | 20180603180140 |
| 1013701W8291H014S | 3003403396115376 | 39.947195°N | 116.121368°E | 20180603211910 |

culated the proportion of mobile phone users in this data to all mobile phone users in each administrative unit's census data to confirm the validity of the data.

Residential population and employed population were calculated based on the mobile phone data. First, we traced the individual people's locations that are most frequently visited during one-month period according to their trajectory information. Second, we recognized the location where a user stayed most on weekends and weekday night time (8:00 p.m. to 5:00 a.m.) during one-month period as his or her place of residence. After then, we recognized the place where he or she stayed mostly day time (5:00 a.m. to 8:00 p.m.) on weekdays during one-month period as the place of work. Third, we aggregated the number of residents to a geographic unit (1 km×1 km grid) and then to a city level in terms of their residence and working place. Fourth, we calculated a weight of a mobile user which he or she can indicate a population size according to the officially reported population number (census). For example, a female mobile user from a given geographical unit in Hengshui (Figure 1) can indicate 2.98 female population. After this step, the number of residential population calculated from mobile phone data is consistent with the number of population reported officially. Fifth, similarly, the number of workers of each geographic unit or city was calculated.

We identified the trips by calculating the trajectory of mobile phone users. A trip refers to move between two places of stays. A stay means a user keeps in one same location, which is positioned for more than 30 min between two timestamps. A trip origin is the departure grid, and a trip destination is the arrival grid. Trips between origin and destination are aggregated as OD (Origin-Destination) flow.

In this study, we aggregated the basic geographic unit (1 km×1 km grid) to a county-level unit which is the primary administrative unit in China's city system (Figure 1). Inter-city mobility is measured by trips flow between the main urbanised area of the county. The main urbanised area refers to the area where land use is mainly occupied by the built-up area or urbanised grids. This can be detected by using land use data from Landsat remote sensing image data. The acqusition time of the remote sensing data is 2018. The formula of inter-city mobility is as follows:

$$V=\sum_{i=1}^{n}\left(\sum_{j=1}^{m}o_{ij}+\sum_{j=1}^{m}d_{ij}\right), \quad (1)$$

where $V$ represents the volume of inter-city travel flow be-

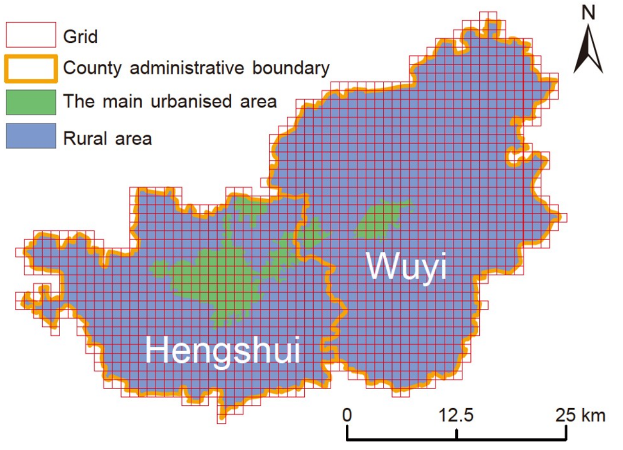

**Figure 1** An example of the main urbanised areas and the county administrative boundary. In this example, the inter-city mobility is the trips between the urbanised area of Hengshui and Wuyi.

tween two cities; $n$ is the number of urbanised grids in one county-level unit; $m$ is the number of urbanised grids in another county-level unit; $o_{ij}$ is the volume of travel flow from grid $i$ to grid $j$ and $d_{ij}$ is the volume of travel flows from grid $j$ to grid $i$.

In this study, residents' travel purposes were divided into commuting, home-based non-commuting, and non-home-based non-commuting. The identification of residents' travel purposes was mainly based on the location information of mobile phone users. According to the users' locations, we speculated on each user's possible residence and workplace. Travel behaviour between residence and workplace was identified as commuting. Travel behaviour between residence and other places was recognised as home-based non-commuting. Travel activities other than the above two types were identified as non-home-based non-commuting. The people aged over 65 are usually retired and people under 18 are usually in school. Therefore, these two groups of people have no commuting trips for work. The users whose work place and home place are in the same grid don't have commuting trips.

## 2.2 Zipf's law

For further analysis of travel volume scale distribution characteristics, we try to verify whether traffic volume follows Zipf's law. Zipf model is a kind of power-law distribution, which can be expressed as follows:

$$\mathrm{Rank}^{\gamma}P^{\delta} = a, \tag{2}$$

$$\gamma \ln(\mathrm{Rank}) + \delta \ln(P) = \ln a. \tag{3}$$

In the formula, $a$ is constant. If $\gamma=\delta$, the distribution follows the standard rank-size law. If $\delta/\gamma>1$, the distribution pattern tends to be dispersed. Otherwise, if $\delta/\gamma<1$, the distribution pattern tends to be concentrated. $P$ and Rank in the formula usually represent the population size of a typical city and its population size rank. For an ideal Zipf's law, $\gamma=\delta=1$ and $a$ equals the population of the largest city.

### 2.3 Gravity models

To analyse the influencing factors of inter-city travel, we established a regression based on a gravity model. A general gravity model is as follows:

$$V = \frac{kO^{\alpha}D^{\beta}}{c^{\theta}}, \tag{4}$$

$$\ln V = \ln k + \alpha \ln O + \beta \ln D - \theta \ln c, \tag{5}$$

where $V$ represents the volume of inter-city travel flow between two cities; $O$ and $D$ the attraction of the origin city and the destination city; $c$ the travel cost between the two cities; and $k$, $\alpha$, $\beta$ and $\theta$ are the coefficients to be calibrated.

Different gravity models are estimated for inter-city travel flow (Tables 2 and 3). All variables can be divided into two parts: the attraction of cities and the impedance between cities. For the attraction part, we provided three options for indicators: residential population, employed population and GDP to test which indicator is most efficient. Residential population and employed population were calculated based on mobile phone data. The details of the calculation is introduced in section 2.1. The data of GDP of each city are from the statistical yearbook of China.

The impedance part consists of travel cost and job-housing balance. For travel cost, we provided two options: Euclidean distance and driving time. The Euclidean distance and driving time were calculated via the Baidu Map API. We used free-flow car-driving time at 10 am to measure driving time. Job-housing balance is indicated by the ratio of residential population to employed population of two cities.

We estimated eight gravity models in order to confirm which variables are more suitable to represent attraction and travel cost (Table 3). For the attraction part of the model, Models 1, 2 and 3 were compared to find the most efficient indicator from residence, employees and GDP. For the travel cost part, Models 1 and 4 were compared to find the more efficient indicator from Euclidean distance and driving time. Similarly, Models 2 and 5 and Models 3 and 6 were estimated to find the more efficient indicator for travel cost. In addition, Model 7 and 8 were estimated to check whether job-housing balance is an efficient variable in the model.

Firstly, Models 4, 5 and 6 are better than Models 1, 2 and 3. As other variables are controlled, Models 1, 2 and 3 use Euclidean distance to indicate travel costs, while Models 4, 5 and 6 use driving time. If we compare Model 1 with Model 4, Model 2 with Model 5, and Model 3 with Model 6, we find that the latter ones' adjusted $R$ squared is higher.

Secondly, Models 4 and 5 are better than Model 6. The effectiveness of Models 4 and 5 is almost the same, but they are obviously better than that of Model 6. Models 4 and 5 use population to indicate attraction. If we compare Model 6 with Models 4 and 5, we find that population is more efficient than GDP. Then, if we compare Model 4 with Model 5, we find that employment population is slightly more efficient than residential population.

Thirdly, Models 7 and 8 are optimisations of Models 4 and 5. Models 7 and 8 have similar overall effectiveness. The difference is that all the variables in Model 7 are significant.

## 3. Results

### 3.1 Travel purposes of inter-city mobility

Figure 2 shows the proportion of inter-city mobility by travel purpose. For intracity travel, commuting accounts for one fourth of total trips on weekdays. However, for inter-city travel, commuting occupies a very small proportion of total trips on weekdays, only 12%. Home-based non-commuting travel, which includes the trips between living place and nonworking place, dominates inter-city travel. It accounts for 62% of inter-city trips. This situation does not vary significantly between weekdays and weekends. The proportion

**Table 2** Indicators to be tested in the model

| Variable in the general gravity model | Factor | Indicator of different options | Abbreviation |
|---|---|---|---|
| $O$ and $D$ | Attraction | Option 1: Residential population | $\mathrm{Pop}_{\mathrm{re}}$ |
| | | Option 2: Employed population | $\mathrm{Pop}_{\mathrm{em}}$ |
| | | Option 3: GDP | GDP |
| $c$ | Impedance 1: Travel cost | Option 1: Euclidean distance | $\mathrm{Cost}_{\mathrm{dis}}$ |
| | | Option 2: Driving time | $\mathrm{Cost}_{\mathrm{time}}$ |
| | Impedance 2: Job-housing balance | Ratio of residential population to employed population | JHB |

**Table 3** Results of regression based on the gravity model[a)]

| | Model 1 | Model 2 | Model 3 | Model 4 | Model 5 | Model 6 | Model 7 | Model 8 |
|---|---|---|---|---|---|---|---|---|
| Constant | −7.158*** | −5.602*** | 6.841*** | −4.358* | −2.88 | 9.186*** | 1.173 | 1.173 |
| Standard error | −2.196 | −2.024 | −1.42 | −2.207 | −2.055 | −1.468 | −3.366 | 3.366 |
| $Pop_{re}$ | 0.764*** | | | 0.733*** | | | 0.690*** | |
| Standard error | −0.063 | | | −0.06 | | | −0.062 | |
| $Pop_{em}$ | | 0.756*** | | | 0.725*** | | | 0.690*** |
| Standard error | | −0.06 | | | −0.058 | | | 0.062 |
| GDP | | | 0.557*** | | | 0.539*** | | |
| Standard error | | | −0.056 | | | −0.052 | | |
| $Cost_{dis}$ | −1.455*** | −1.451*** | −1.391*** | | | | | |
| Standard error | −0.156 | −0.153 | −0.178 | | | | | |
| $Cost_{time}$ | | | | −1.827*** | −1.816*** | −1.797*** | −1.802*** | −1.802*** |
| Standard error | | | | −0.177 | −0.174 | −0.198 | −0.173 | 0.173 |
| JHB | | | | | | | −2.464** | −1.774 |
| Standard error | | | | | | | −1.151 | 1.173 |
| $R$ squared | 0.802 | 0.809 | 0.748 | 0.824 | 0.829 | 0.783 | 0.834 | 0.834 |
| Adjusted $R$ squared | 0.797 | 0.804 | 0.742 | 0.819 | 0.824 | 0.777 | 0.827 | 0.827 |

a) ***$p$≤0.001, **$p$≤0.01, *$p$≤0.05

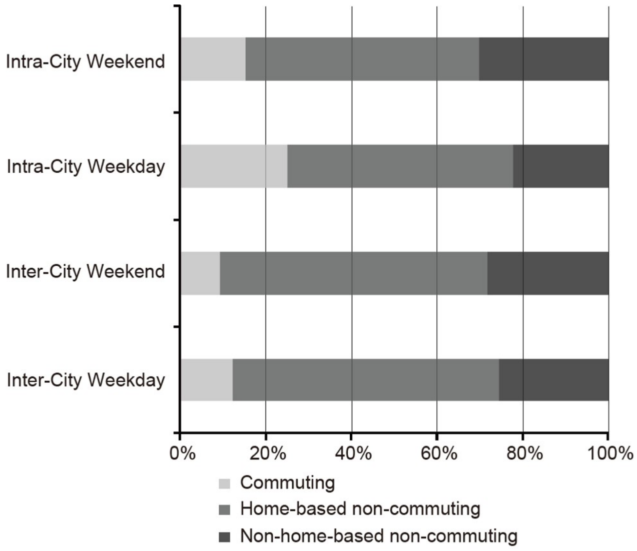

**Figure 2** Percentage of travel purposes in different periods. The travel purposes are identified based on the location information of mobile phone users. Travel behaviour between residence and workplace was identified as commuting. Travel behaviour between residence and other places was recognised as home-based non-commuting. Travel behaviour other than the above two types was identified as non-home-based non-commuting.

of commuting increases from 9% on weekends to 12% on weekdays, while the proportion of non-commuting trips is very similar on weekdays and weekends. Non-home-based non-commuting trips, which refer to trips where neither the place of departure nor the place of destination is a place of residence or a place of work, account for 26.5% on average. Interestingly, the proportion of non-home-based trips is higher on weekends, 28%, than on weekdays, 25%.

### 3.2 Travel distance for inter-city mobility

We have calculated and visualised the spatial pattern of inter-city travel volume (Figure 3a). The figure shows that inter-city trips are mainly concentrated in the region's central area around the biggest city. Figure 3b shows the average travel distance per traveller in each grid. The average distance of inter-city travel is 64.43 km. Trips with a distance between 30 and 50 km accounted for about 21.75% of trips. It is notable that the travel distance for residents in urban central areas is slightly higher than that in other areas. The travel distance for residents in mountainous areas in the northwest region is also higher. Figure 3c shows that there is a negative correlation between the number of travellers and the distance between cities, which is consistent with gravity models.

However, there are large differences in inter-city travel distance for different travel purposes (Figure 4). Commuting involves the shortest distance of the three travel purposes, 43.47 km on average. The average inter-city travel distance for non-commuting travel is 62.24 km. Non-home-based non-commuting travel has the longest distance, 69.59 km. The most common distance for home-based travel, both commuting and non-commuting, is less than 10 km, while the most common distance for non-home-based travel is around 50 km. This means that people are willing to travel longer between cities for non-commuting purposes than for commuting.

Furthermore, we investigated the relationship between inter-city travel distance and city size (Figure 5). We found that there is a positive correlation between travel distance

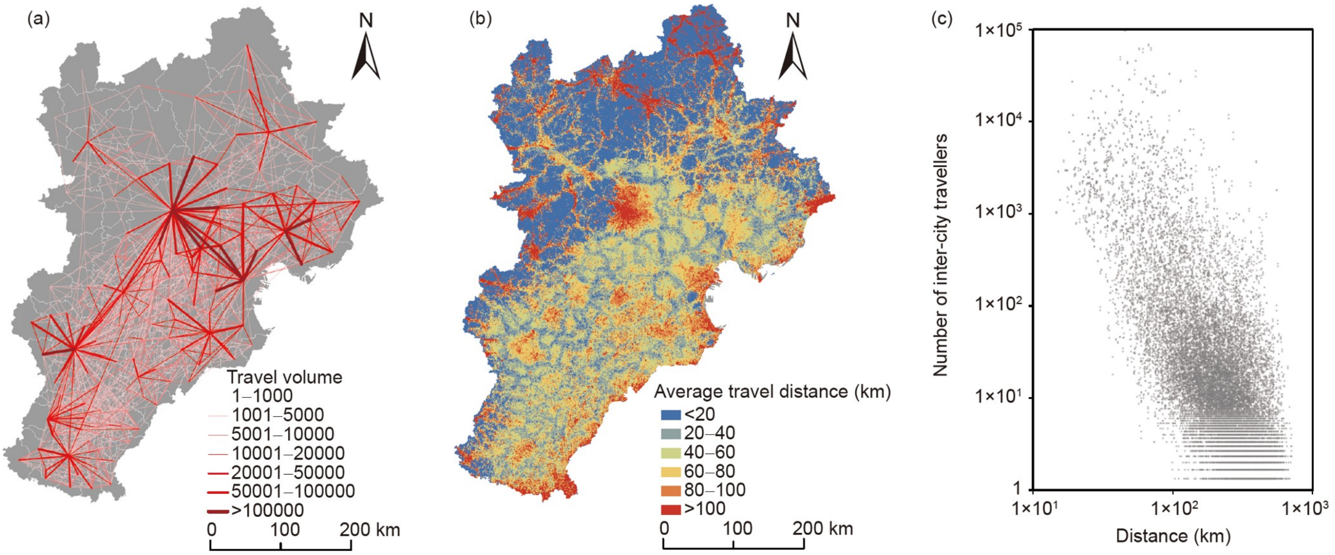

**Figure 3** Inter-city travel volume and travel distance distribution in the Jing-Jin-Ji megacity region. (a) Visualization of travel volume between county-level cities; (b) distribution of average travel distance of travellers in each 1 km grid; (c) scatter plot of the number of inter-city travellers and the inter-city distance. Each point represents inter-city trips between two cities.

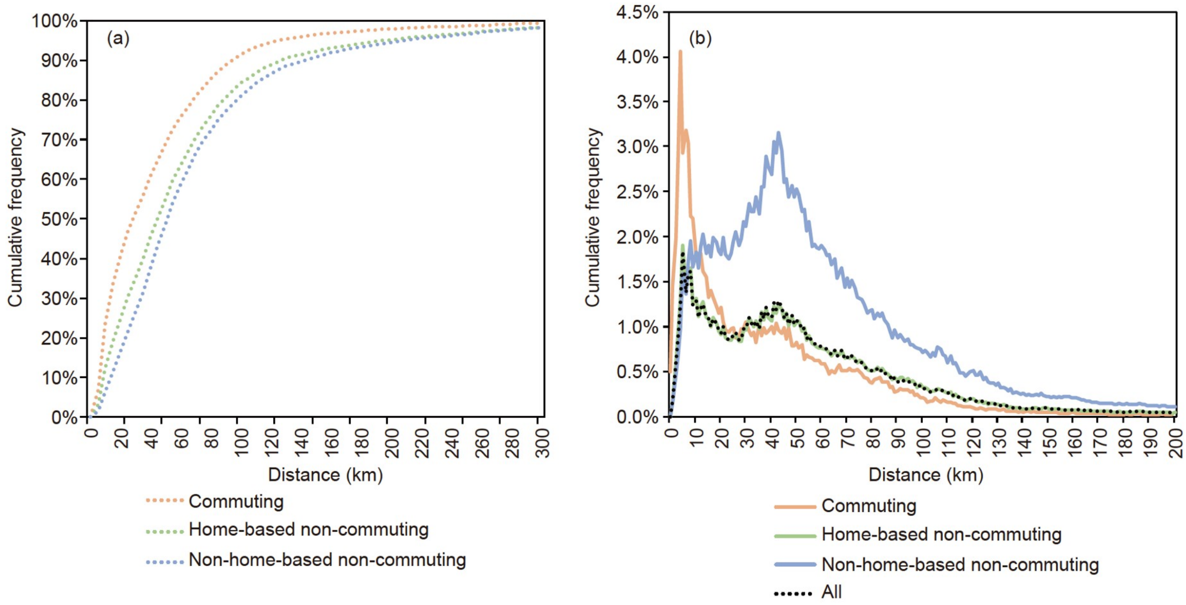

**Figure 4** Travel distances for different purposes. (a) Line plots showing cumulative frequency of different travel distances for different purposes; (b) line plots showing the proportion of different travel distances for different purposes.

and city size as a whole. If we classify these cities according to the level of job-housing balance, we will find more interesting internal differences. For cities in which the employed population is greater than the residential population, inter-city travel distance tends to increase significantly with the expansion of city size. These cities are mainly employment centres, including Beijing, the largest city in the region. For these employment centres, the larger city size can attract residents from further away to commute there. For cities with balanced employment and living, the inter-city travel distance is also positively related to the city size. However, for cities in which the residential population is greater than the employment population, there is a negative correlation between inter-city travel distance and urban size, but the trend is not significant. Most of these cities are small cities with populations of less than 500,000. The travel distance of these cities varies greatly, and the difference among them may be determined by other factors.

### 3.3 Hierarchical structure law of inter-city mobility

Zipf's law was established first to explain word frequency

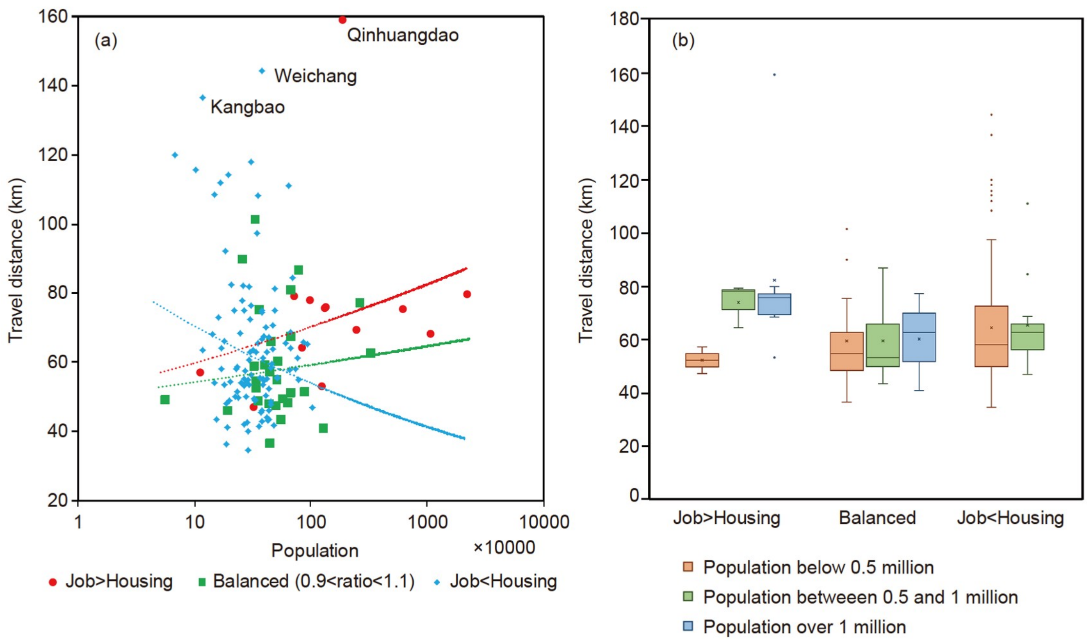

**Figure 5** Travel distance and city size. (a) Scatter plots show positive correlation of inter-city travel distance with city population size while the employed population is not smaller than the residential population. The correlation is negative while the residential population is greater than the employed population, but the trend is not significant. (b) The average travel distance differs between different population groups.

patterns in context in the 1930s (Zipf, 1937). Zipf's law as applied to an urban system states that the expected population size of a city relates to its rank in a size table (Yaqoob et al., 2016). In this study, we use the Zipf curve to identify whether there is a hierarchical structure of inter-city mobility. We found that the coefficient is very close to 1 (Figure 6). This means that both travel volume and city size in the whole region basically follow Zipf's law.

However, there are still discrepancies. The coefficient is greater than 1 for travel volume, while smaller than 1 for population. This reflects that the distribution of travel volume is not as centralised as the population. The results are as follows:

$$\mathrm{Rank}_{\mathrm{pop}} = \frac{8.37\times10^{6}}{\mathrm{Pop}^{1.0434}}, \tag{6}$$

$$\mathrm{Rank}_{\mathrm{mobility}} = \frac{3.63\times10^{6}}{\mathrm{Mobility}^{0.9835}}. \tag{7}$$

Why does inter-city travel volume follow a more dispersed distribution? We separately analyse 13 major cities, which are the largest cities and administrative centres of each prefecture. The goodness of fit is better when we only take into account 13 major cities than when we take all the cities. The relationship between travel volume and city rank among the 13 major cities follows Zipf's law well, as shown in the bottom half of Figure 6. However, the Zipf curve of travel volume is much steeper than that of the general population. The coefficients are 1.4018 and 0.6604, respectively, which means that the degree of deviation from the standard Zipf distribution is greater than those of all cities. This result shows that compared with the prominent difference in population size of these major cities, their difference in inter-city travel volume is smaller. It proves that big cities are mainly causing discrepancies.

We examined different travel purposes separately. The results showed that although both commuting and non-commuting basically follow Zipf's law (Figure 7), the coefficient for non-commuting travel (0.868) is lower and further from 1 than for commuting travel (1.06). The results are as follows:

$$\mathrm{Rank}_{\mathrm{commuting}} = \frac{1.95\times10^{5}}{\mathrm{Commuting}^{0.868}}, \tag{8}$$

$$\mathrm{Rank}_{\mathrm{Non\text{-}commuting}} = \frac{9.15\times10^{6}}{\mathrm{Non\text{-}commuting}^{1.0601}}. \tag{9}$$

This reflects that the inter-city commuting hierarchy is more concentrated than that of non-commuting travel behaviours. Specifically, large cities' advantages over small cities in inter-city commuting travel volume are greater than that in inter-city non-commuting travel volume. Inter-city commuting takes place far more frequently between top-ranking cities than between low-ranking cities, while the differences in non-commuting behaviour between top-ranking cities and low-ranking cities are not so prominent. We find that the distributions of both overall inter-city trip and non-commuting inter-city trips are relatively close to the standard Zipf distribution. Only the distribution of inter-city commuting is more concentrated in larger cities.

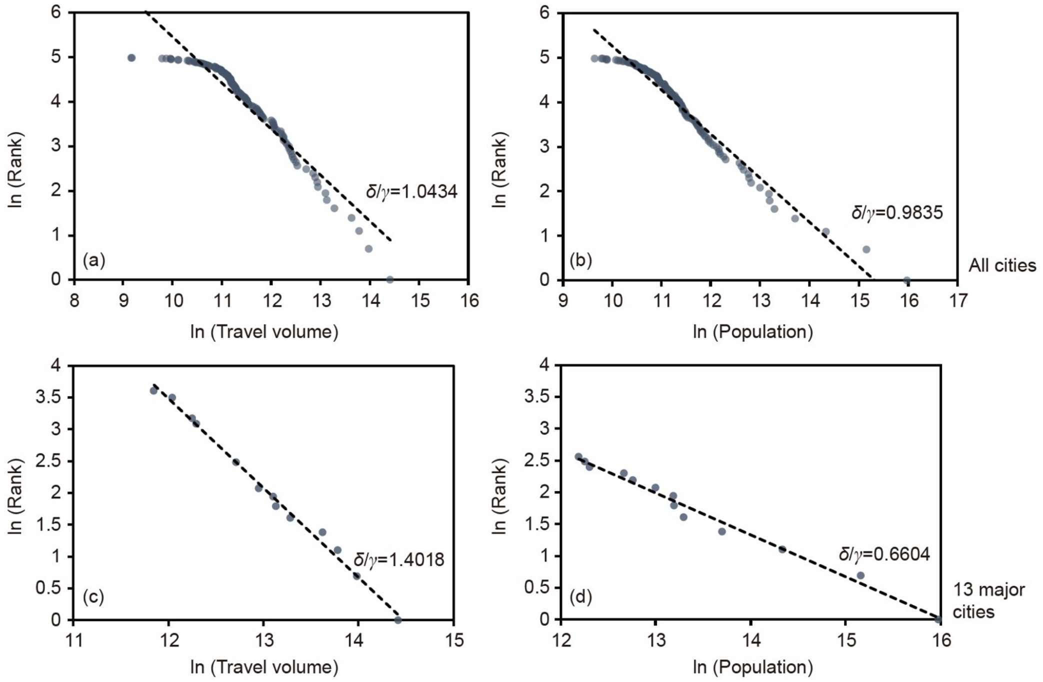

**Figure 6** Zipf curve of inter-city travel volume and population. (a) Rank-size distribution of inter-city travel volume of each city. (b) Rank-size distribution of city population. (c) and (d) show the rank-size distribution of travel volume and population of 13 major cities. All data are consistent with Zipf's law which are well approximated by a power law.

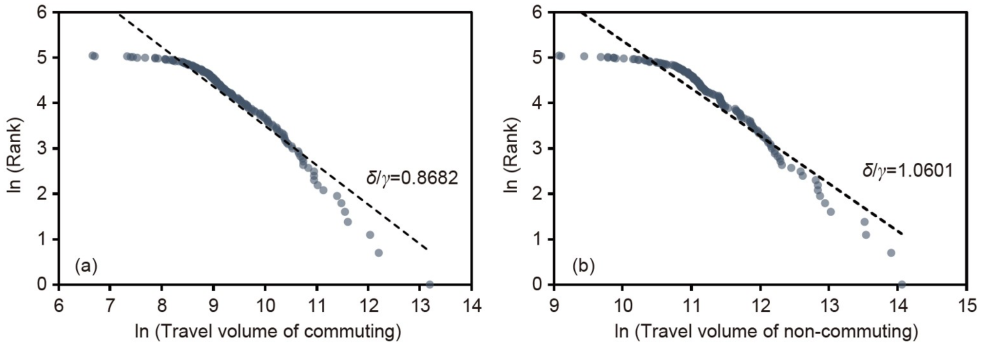

**Figure 7** Zipf curve of inter-city travel volume separated by travel purposes. (a) Rank-size distribution of inter-city commuting volume of each city. The data can be approximated by a power law with exponent −0.8682. (b) Rank-size distribution of inter-city non-commuting volume of each city. The data can also be approximated by a power law and the exponent is −1.0601.

### 3.4 Spatial distribution law for inter-city mobility

It is widely believed that spatial distribution of inter-city mobility follows a gravity model law (Mayo et al., 1988), which means the travel volume between two cities is positively related to their population or economic size and negatively related to travel costs between these cities.

We use mobile phone trajectory big data to estimate a gravity model for inter-city mobility. To obtain a better model, we use different indicators to describe the attraction of cities and the impedance between cities in the gravity model. For the attraction part, we use residential population, employed population and GDP as the indicators. We do not use these indicators at the same time, because we find that there is serious collinearity between them through VIF testing. By comparing the goodness of fit, we find that the employed population is the best indicator of the attractiveness of cities. For the impedance part, we discovered that driving time cost is the better indicator according to a comparison of the goodness of fit. Therefore, the model is as follow:

$$V = \frac{0.056\mathrm{Pop}_{\mathrm{em}}^{0.725}}{\mathrm{Cost}_{\mathrm{time}}^{1.816}}. \tag{10}$$

On this basis, although the simultaneous inclusion of population size and economic size into the model causes collinearity problems, we still want to investigate the impact of job-housing balance on inter-city travel. We are pleasantly surprised to find that job-housing balance is a significant factor in inter-city travel. This indicates that the higher the ratio of housing to a job in a city, the less the inter-city travel volume tends to be. The optimised model is as follows:

$$V=\frac{3.232\mathrm{Pop}_{\mathrm{re}}^{0.690}}{\mathrm{Cost}_{\mathrm{time}}^{1.802}\mathrm{JHB}^{2.464}}. \tag{11}$$

Furthermore, we estimated gravity models for weekday and weekend. The results show that inter-city travel on weekdays is more affected by travel time and job-housing balance than weekends. The optimised models are as follows:

$$V_{\mathrm{weekday}}=\frac{3.565\mathrm{Pop}_{\mathrm{re}}^{0.702}}{\mathrm{Cost}_{\mathrm{time}}^{1.834}\mathrm{JHB}^{3.035}}, \tag{12}$$

$$V_{\mathrm{weekend}}=\frac{0.145\mathrm{Pop}_{\mathrm{re}}^{0.693}}{\mathrm{Cost}_{\mathrm{time}}^{1.705}\mathrm{JHB}^{1.416}}. \tag{13}$$

There are also differences in the gravity model when different travel purposes are considered. As in the method described above, we tested different indicators to find better models for three kinds of inter-city travel behaviour: commuting, home-based non-commuting and non-home-based non-commuting. The optimised models are as follows:

$$V_{\mathrm{commuting}}=\frac{62245\mathrm{GDP}^{0.778}}{\mathrm{Cost}_{\mathrm{time}}^{1.923}\mathrm{JHB}^{4.450}}, \tag{14}$$

$$V_{\text{home-based non-commuting}}=\frac{6.998\mathrm{Pop}_{\mathrm{re}}^{0.642}}{\mathrm{Cost}_{\mathrm{time}}^{1.765}\mathrm{JHB}^{2.551}}, \tag{15}$$

$$V_{\text{non-home-based non-commuting}}=\frac{0.012\mathrm{Pop}_{\mathrm{em}}^{0.753}}{\mathrm{Cost}_{\mathrm{time}}^{1.848}}. \tag{16}$$

The three models produce effective prediction results (Figure 8). Comparing these three models, we find several differences among them. Firstly, for these three travel purposes, different indicators are appropriate to represent the attraction of cities in gravity models. The best indicator for the attraction of inter-city commuting is GDP, while the best indicator for attraction of inter-city non-commuting is population. In non-commuting, the attraction of home-based travel is a better representation residential population, while that of non-home-based travel is indicated more accurately by the employed population. Secondly, job-housing balance is a significant indicator for home-based travel, both commuting and non-commuting, while it is not a significant indicator for non-home-based travel. In home-based travel, job-housing balance has a greater impact on commuting than on non-commuting.

## 4. Discussion and conclusions

Human beings are ushering in an urban age (Angel et al., 2011). Megacity regions, where many megacities interact closely and over 100 million people reside, are becoming one

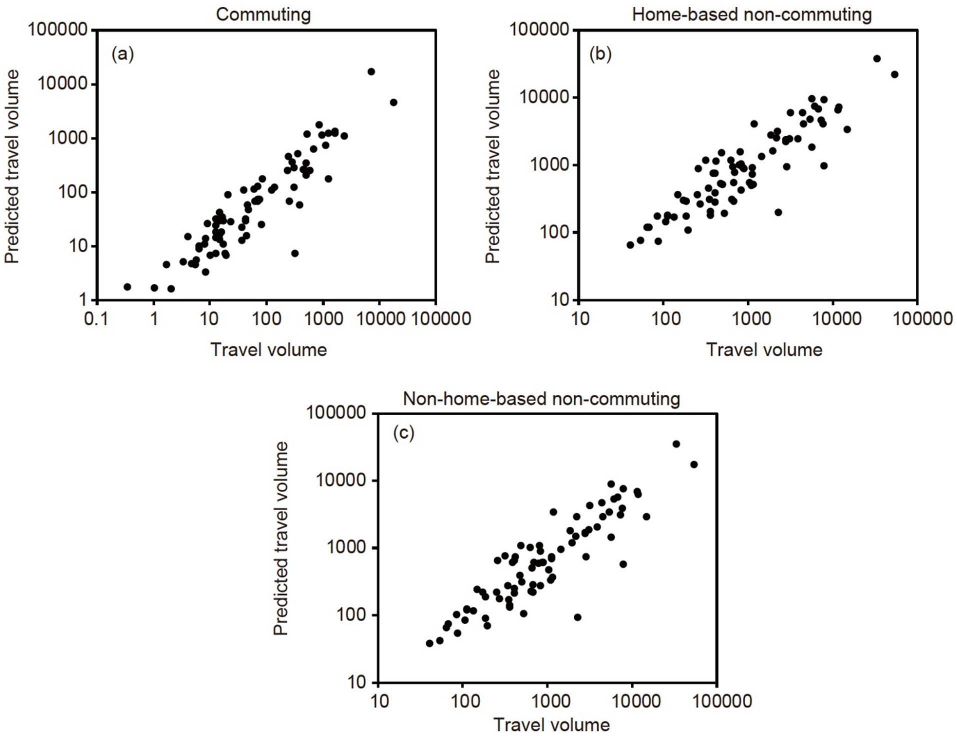

**Figure 8** Prediction of inter-city travel volume based on the gravity model. (a)–(c) respectively shows the predicted value and true value of inter-city travel volume for different purposes, including commuting, home-based non-commuting and non-home based non-commuting.

of the primary forms of human settlement (United Nations, 2019). We contribute a new understanding of the gravity laws of inter-city mobility in a megacity region by examining 40.48 million travellers' trajectories recorded by mobile phones. A number of eight gravity models were measured by testing different key variables including population, employment, GDP, and travel times. The differences between different travel purposes and travel time periods were also analysed.

Firstly, non-commuting travel, rather than commuting, is the primary type of inter-city travel in this megacity region. In large regions, commuting behaviour between cities has received much more attention than non-commuting travel (Lehmer and Möller, 2008; Frederick and Gilderbloom, 2018). It was found that more satisfying jobs or housing in different cities leads to inter-city travel in megacity regions (Hanson and Pratt, 1992; Clark and Dieleman, 1996). But our analysis of the results shows that non-commuting occupies the major proportion of inter-city travel in the Jing-Jin-Ji megacity region. We also found that non-commuting travellers have a travel distance that is 42.3% longer than commuting travellers. In addition, people in large cities have a higher proportion of non-commuting trips and longer travel distances than those who live in small cities.

Secondly, when it comes to city size, there is a hierarchical structure rule of inter-city mobility, namely, the distribution of both the city size and its travel volume follows Zipf's law. It has been reported that the travel radius of mobility follows a power law from an aggregated perspective (Alessandretti et al., 2020). According to our findings, the distribution of inter-city travel volume is relatively more dispersed than that of the population. Previous studies reported that the overall exponent of Zipf's law in different countries is usually close to 1 (Heppenstall et al., 2011). However, if cities are divided into several parts according to size, the exponents of the best-fit power law that describes each part tend to be different.

In addition, our research was the first to observe the differences in Zipf's law for commuting and non-commuting travel. We ascertained the difference in their exponents and confirmed that the exponent of Zipf's law for commuting is smaller than that for non-commuting. This shows that the larger the city size, the more likely inter-city commuting is to occur, and this possibility is growing faster than that of non-commuting.

Thirdly, the spatial distribution of inter-city trips for different purposes follows different forms of gravity models. It is widely believed that trips between different geographical locations within one city follow the gravity model (Erlander and Stewart, 1990). How about trips between different cities in a megacity region? Our findings provide an answer for this question. We found that a hybrid model integrating both population and job-housing balance variable is more efficient than a model that only includes population or GDP, since the hybrid model can give a more accurate travel volume prediction. Commuting and non-commuting inter-city trips suit different gravity models. One of the major reasons why the models are slightly different is that different travel purposes represent different travel behaviour and social-economical mechanisms. The occurrence of commuting is closely related to the job market and the housing market. GDP reflects the economic volume of a city, thus reflecting the size and attractiveness of its job market. Job-housing balance affects whether all residents can find jobs locally and then affects the possibility of inter-city commuting. Oppositely, non-commuting travel purposes are more extensive. The difference in public service facilities in each city is not as big as that in the levels of economic development, so the supply of urban public service facilities is more closely related to the size of the urban population, which makes the residential population a better variable to indicate attraction in the model. Non-home-based travel mainly includes business trips, so the employed population is a better source to indicate attraction.

Fourthly, big-data analytics have a high value when examining inter-city trips in a megacity region with a large geographical scale and millions of residents. It is worthy of wide application. Compared with the travel survey, mobile phone trajectory big data gives more accurate and complete travel chains at a lower human and financial cost. The base station can obtain the location of the user in real time, so all the location information of the user can be captured to form complete travel chains. Therefore, the influence of residents' memory errors in travel survey is avoided. At the same time, this location acquisition technology is consistent on a large geographical scale, as it is run by the same operator, which avoids any inconsistency of standards in manual investigation. We also need to recognise that big data is a double-edged sword, and that privacy protection is an issue that needs attention in future big-data applications. In this study, the user's trips are aggregated on the grid, so the user's personal information is not involved, which complies with the principle of privacy protection.

**Acknowledgements** *We thank colleagues of the Centre for Urban Planning and Transport Studies, Peking University for giving advice to this work. This work was supported by the National Natural Science Foundation of China (Grant Nos. 41925003 & 42130402) and the Beijing Municipal Social Science Foundation (Grant No. 20JCB073).*

**Open Access** This article is licensed under a Creative Commons Attribution 4.0 International License, which permits use, sharing, adaptation, distribution and reproduction in any medium or format, as long as you give appropriate credit to the original author(s) and the source, provide a link to the Creative Commons licence, and indicate if changes were made. The images or other third party material in this article are included in the article's Creative Commons licence, unless indicated otherwise in a credit line to the material. If material is not included in the article's Creative Commons licence and your intended use is not permitted by statutory regulation or exceeds the permitted use, you will need to obtain permission directly from the copyright holder. To view a copy of this licence, visit http://creativecommons.org/licenses/by/4.0/.
